# Supplementary material for: Functional connectivity-based attractor dynamics of the human brain in rest, task, and disease
Source: eLife. 2026 Mar 18;13:RP98725. doi: 10.7554/eLife.98725 (PMC12999174; doi:10.7554/eLife.98725)
Supplement: Figure 4—source data 1. [file elife-98725-fig4-data1.docx]

**Figure 4-source data 1. Neurosynth meta-analyses.** The table includes details about the term used for the automated meta-analyses, as well as the number of studies included in the meta-analysis, the total number of reported activations and the maximal Z-statistic from the meta-analysis.

| search term | num. studies | num. activations | max. Z |
| --- | --- | --- | --- |
| pain | 516 | 23295 | 14.8 |
| motor | 2565 | 109491 | 22.5 |
| auditory | 1252 | 46557 | 25.3 |
| visual | 3110 | 115726 | 15.4 |
| face | 896 | 31842 | 26.8 |
| autobiographical | 143 | 7251 | 15.7 |
| theory of mind | 181 | 7761 | 15.1 |
| sentences | 356 | 13461 | 16.5 |
